# Achieving Hemostasis With a Foley Catheter for Hemorrhagic Shock Caused by an Aortoesophageal Fistula of an Infected Thoracic Aortic Aneurysm With Dissection: A Case Report

**DOI:** 10.7759/cureus.71882

**Published:** 2024-10-19

**Authors:** Taiken Banno, Masaru Shimizu, Misayo Nishikawa

**Affiliations:** 1 Anesthesiology, Uji Tokushukai Medical Center, Uji, JPN

**Keywords:** aortoesophageal fistula, foley catheter, hematemesis, infected thoracic aortic aneurysm, life-saving procedures

## Abstract

Aortoesophageal fistulas (AEFs) are a rare complication of thoracic aortic aneurysms. Moreover, the associated massive bleeding from the upper gastrointestinal tract results in a very high mortality rate. In this report, we describe a successful experience in using a Foley catheter to save a patient’s life. A 46-year-old man was admitted for an infected thoracic aortic aneurysm with dissection. Seven days after admission, emergency thoracic aortic stent graft insertion was performed due to esophageal perforation of the thoracic aortic aneurysm. Three hours after starting surgery, a diagnosis of continuous fresh blood hematemesis and an acute exacerbation of an AEF associated with an infected thoracic aortic aneurysm was established. The patient’s blood pressure was no longer maintained due to massive bleeding. We temporarily placed a Foley catheter in the middle esophagus. Hemostasis with a Foley catheter was temporary and stopped the bleeding out of the body. The patient required intensive care to control the bleeding into the left thoracic cavity, and veno-venous extracorporeal membrane oxygenation had to be instituted for the worsening oxygenation. Increased intra-esophageal pressure due to balloon tamponade was considered a possible postoperative complication. The patient faced several complications and challenges during the intraoperative management, but a prompt response to these complications and challenges saved the patient’s life. The efficacy of a Foley catheter in rapidly and effectively responding to active bleeding in critical settings was reaffirmed as a crucial aspect of life-saving procedures.

## Introduction

Aortoesophageal fistulas (AEFs) are caused by a rupture of the aorta, ingestion of a foreign body, or advanced malignancy, resulting in communication between the aorta and esophagus. This leads to bleeding from the aorta into the esophagus and the spread of infection from the esophagus into the mediastinum, which can result in further complications. AEFs of thoracic aortic aneurysms have an incidence of 0.1%. They can be fatal if massive bleeding from the aorta into the esophagus occurs [1]. Therefore, prompt diagnosis and treatment are necessary. The operative mortality due to open repair is 55%, despite advances in surgical techniques [2]. There are few reports of life-saving treatment of an AEF of an infected thoracic aortic aneurysm with dissection. Although patients with acute bleeding caused by AEFs can be adequately treated in an emergency with thoracic endovascular aortic repair (TEVAR), stopping bleeding may not be possible sometimes. Hemostasis may not be possible with TEVAR because it does not completely prevent AEF, but its incidence is unknown. One treatment option for emergency hemostasis is the use of the Foley catheter, a method that has been in use for many years. This approach is non-invasive, quick to implement, and can be inserted into the esophagus or bladder and uses the internal pressure of the balloon at the tip to physically pressure the bleeding and temporarily control it [3-5]. In such rare cases, emergency Foley catheter intervention can successfully save the life of patients with a hemorrhagic AEF caused by an infected thoracic aortic aneurysm with dissection. However, this is a temporary treatment, and after stabilizing the patient, one must move on to more radical treatments. The strength of this case is that it confirms that the Foley catheter, despite being an old technology, could be an effective device in achieving immediate hemostasis in emergency situations. Particularly in cases of unexpected massive bleeding, this method can play an important role as a quick and effective first medical management.

This article was previously presented as a meeting abstract at the 43rd Annual Meeting of the Japanese Society of Clinical Anesthesia on December 8, 2023. The patient provided written informed consent for the publication of this case report and its accompanying images.

## Case presentation

**Figure 1:**
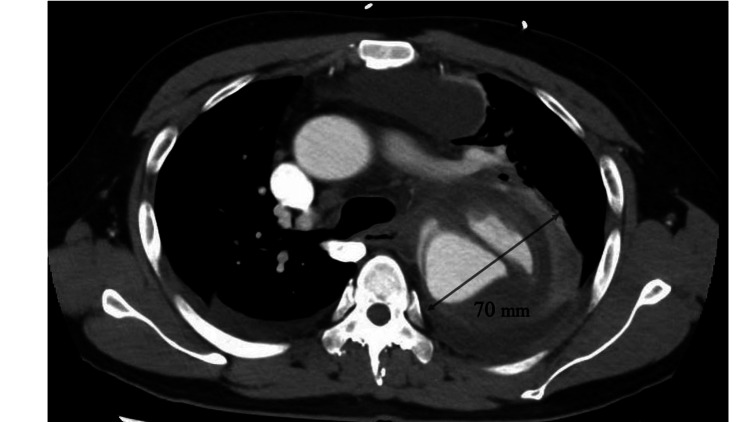
Initial imaging study of the chest Contrast-enhanced chest CT finding showing a 70-mm aneurysm in the distal aortic arch with dissection extending from the distal aortic arch to the common iliac artery. CT, computed tomography

A 46-year-old male had intellectual disability and hypertension. He was administered antipsychotics and antihypertensive medications. He had fever with prothoracic pain for seven days and dyspnea for one day prior to admission and visited a clinic owing to persistent symptoms. Thoracoabdominal contrast computed tomography (CT) showed a 70-mm aneurysm in the distal arch aorta and aortic dissection extending from the distal arch aorta to the common iliac artery (Figure 1).

The patient was diagnosed with an infected thoracic aortic aneurysm with dissection and imminent rupture and transported to our hospital for treatment. The patient had renal dysfunction and an elevated inflammatory response on blood tests after admission (Table 1).

**Table 1:** The blood tests on initial presentation at our hospital. PT-INR, Prothrombin Time-International Normalized Ratio; APTT, Activated Partial Thromboplastin Time

Test	Results	Units	Normal Range
Complete Blood Count			
White Blood Cells	235	× 10^2^/µL	33 - 86
Hemoglobin	11.8	g/dL	13.7 - 16.8
Hematocrit	34.1	%	40.7 - 50.1
Platelet	14.6	× 10^4^/μL	15.8 - 34.8
Coagulation Tests			
PT-INR	1.26		0.9 - 1.1
APTT	31.2	seconds	24 - 34
Fibrinogen	697	mg/dL	200 - 400
Biochemistry			
C-Reactive Protein	29.54	mg/dL	-0.14
Creatine Phosphokinase	9	U/L	59 - 248
Creatine Kinase Muscle-Brain	1	U/L	4 - 16
Sodium	133	mmol/L	138 - 145
Potassium	3.8	mmol/L	3.6 - 4.8
Chloride	99	mmol/L	101 - 108
Urea Nitrogen	21.3	mg/dL	8. 0 - 20. 0
Creatinine	2.08	mg/dL	0. 65 - 1. 07
Aspartate Aminotransferase	22	U/L	13 - 30
Alanine Aminotransferase	22	U/L	10 - 42
Lactate Dehydrogenase	215	U/L	124 - 222
Total Bilirubin	2.98	mg/dL	0. 4 - 1.5
Total Protein	5.2	g/dL	6.6 - 8.1
Albumin	2.1	g/dL	4. 1 - 5.1

**Figure 2:**
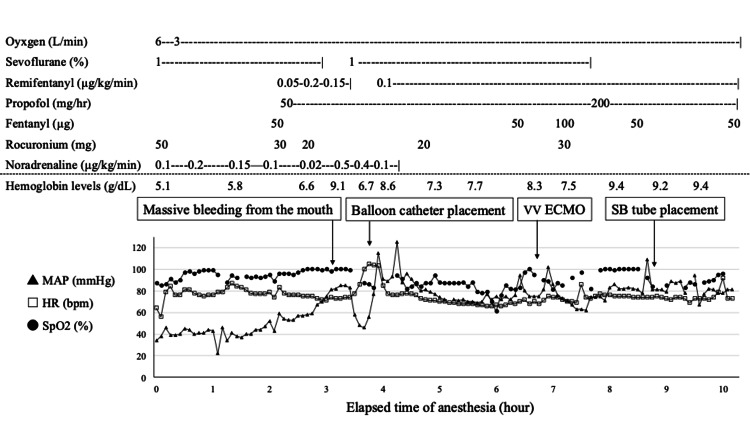
Anesthesia record, operative note, and intraoperative hemoglobin levels. Triangles indicate MAP, squares HR, and circles SpO2. MAP, mean blood pressure; HR, pulse rate; SpO2, peripheral oxygen saturation; VV ECMO, veno-venous extracorporeal membrane oxygenation; SB tube, Sengstaken-Blakemore tube

The patient showed a respiratory rate of 27 breaths/min and a peripheral oxygen saturation of 88% (O2 5L). The patient was intubated for respiratory failure and sedated and managed with blood pressure control and continuous renal replacement therapy. Due to an infected thoracic aortic aneurysm, the patient did not undergo surgery immediately and was treated with meropenem and vancomycin. Seven days after admission, the patient suddenly became hypotensive, and emergency thoracic aortic stent graft insertion due to esophageal perforation of the thoracic aortic aneurysm was planned. Hemoglobin levels were 4.3 g/dL; no hematemesis was noted. After entering the operating room, the patient was anesthetized with 50 mg of rocuronium administered intravenously for induction and sevoflurane (1%) for maintenance (Figure 2).

**Figure 3:**
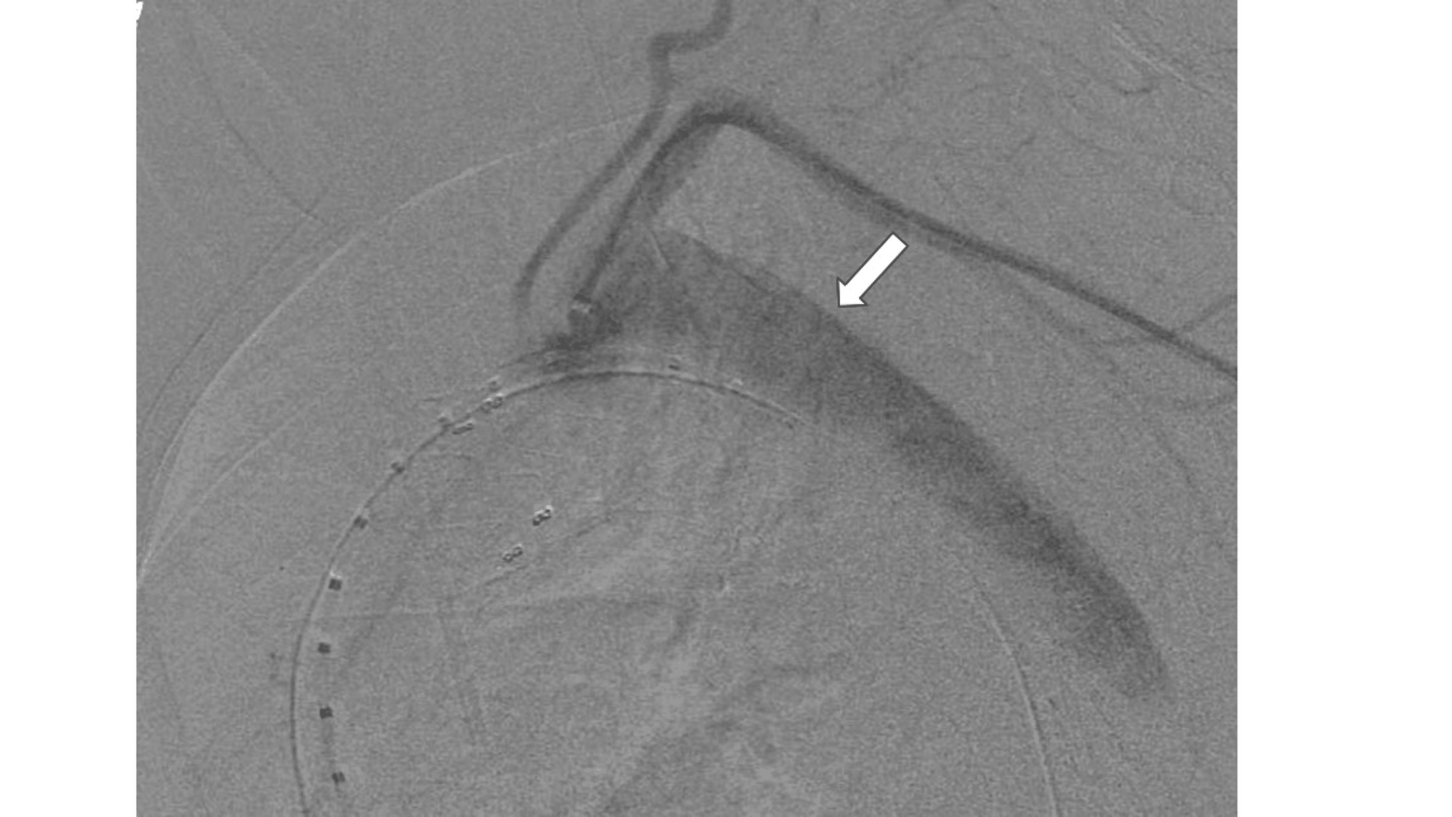
Intraoperative findings of TEVAR. TEVAR can not cover the esophageal perforation area completely and continued false lumen filling (arrow). TEVAR, thoracic endovascular aortic repair

The mean blood pressure was 34 mmHg and the heart rate was 64/min; accordingly, the patient was administered a blood transfusion and noradrenaline. No hematemesis was noted. Three hours after starting surgery, the patient suddenly had intensifying hematemesis and hypotension. Although a blood transfusion was administered and the dose of noradrenaline was increased, the blood pressure did not rise. We were unable to stop the bleeding because the stent graft did not completely attach to the AEF (Figure 3).

**Figure 4:**
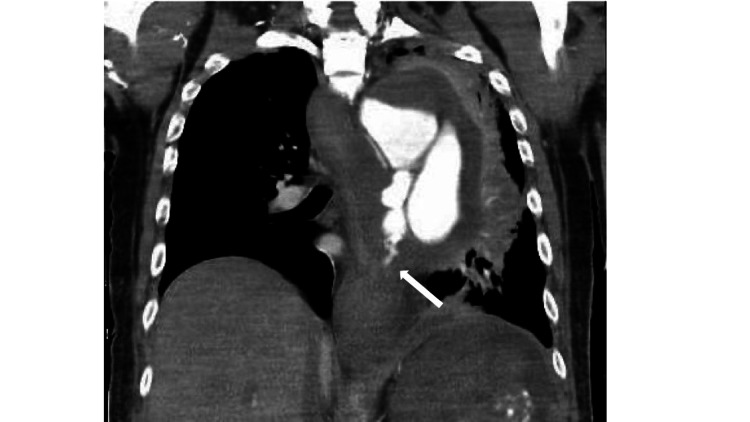
Imaging study of the chest after hospitalization Contrast-enhanced chest CT finding showing approximately 300 mm from the oral cavity to the esophageal perforation (arrow). CT, computed tomography

Therefore, we determined that achieving hemostasis in the patient by managing the esophagus was necessary. Thoracoabdominal contrast CT showed that the AEF was approximately 30 cm from the mouth (Figure 4).

**Figure 5:**
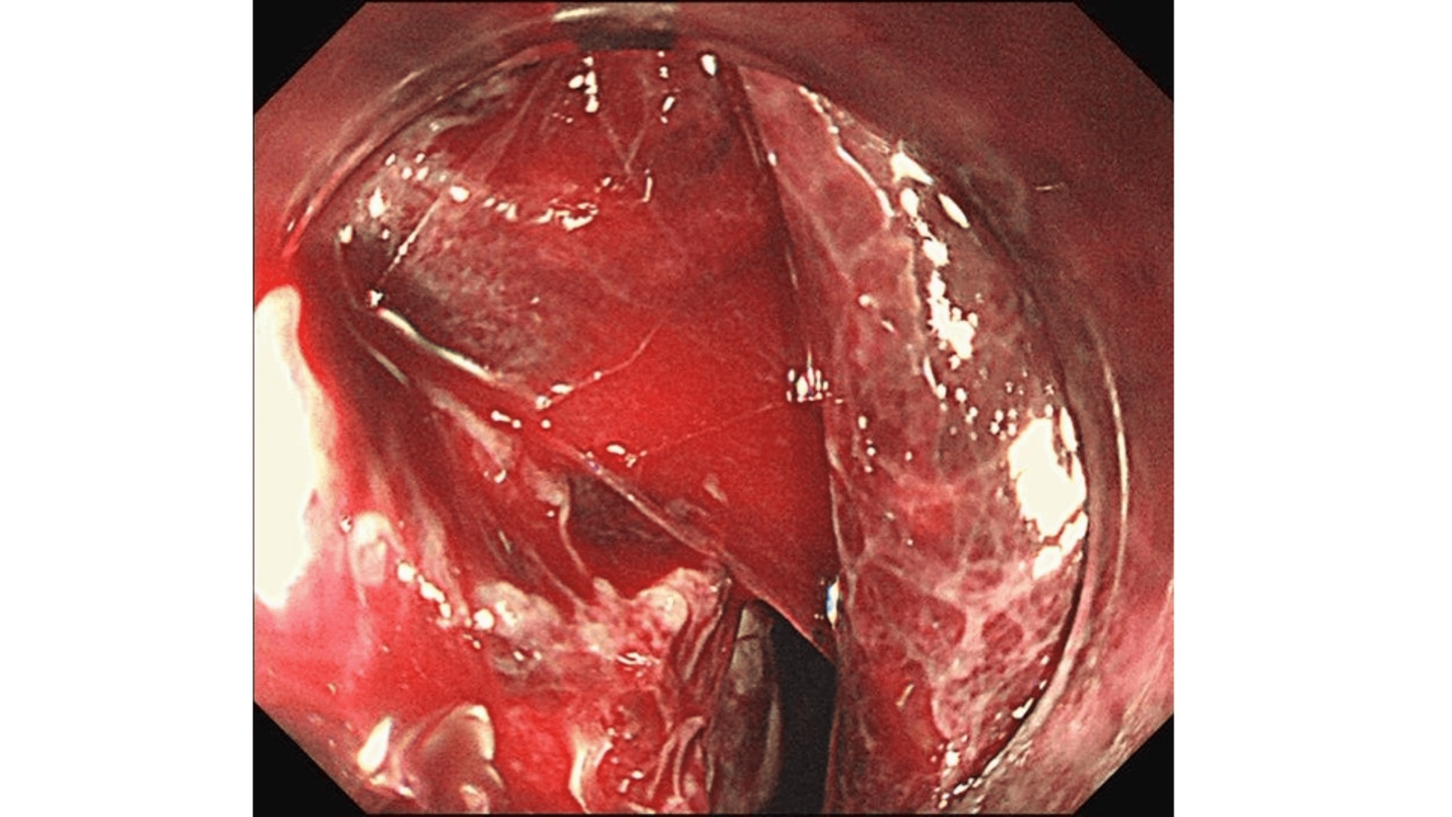
Esophagoscopy findings during operation Esophagoscopy findings showing bleeding into the esophagus, but the site of bleeding is unknown.

We placed a 24-Fr Foley catheter in the middle esophagus 28 cm from his mouth. After Foley catheter placement, bleeding from the mouth decreased. Thereafter, the left subclavian artery was occluded with the coils. His mean blood pressure increased to 70-80 mmHg; noradrenaline was reduced. Oxygenation worsened due to bleeding into the left thoracic cavity and massive transfusions. Accordingly, the patient was treated with veno-venous extracorporeal membrane oxygenation (VV ECMO). We performed upper gastrointestinal endoscopy and confirmed that there was no active bleeding from the AEF (Figure 5).

The Foley catheter was replaced with a Sengstaken-Blakemore tube (SB tube). Estimated blood loss and urine output were 8,000 and 2,200 mL, respectively. A total of 3,000 mL of crystalloid, 1,250 mL of 5% albumin, 9,250 mL of packed red blood cells, 9,600 mL of fresh frozen plasma, and 1,200 mL of platelet concentrate were administered intraoperatively. Postoperatively, the patient was transferred to the intensive care unit on a ventilator and VV ECMO under sedation. Postoperative hemoglobin was 8.1 g/dL. Additional blood transfusions were performed, after which the hemoglobin and fibrinogen measurements were 8 g/dL and 180 mg/dL, respectively. Unfortunately, three months later, the patient died of pneumonia.

## Discussion

AEFs are very rare and difficult to treat when the patient is in hemorrhagic shock, similar to this case [6]. Recently, there have been an increasing number of reports of intraoperative complications with an increase in cases of endovascular aortic repair [7]. AEFs generally result from aortic-related causes, including ruptured thoracic aortic aneurysms and post-prosthetic vascular replacement, accounting for approximately 70% of AEFs [8-10]. Other causes of AEFs include ingestion of foreign bodies, esophageal cancer, and prior aortic surgery [1,11]. There are various mechanisms of AEF formation, including necrosis of the tissue between the arterial and esophageal walls due to inflammation or infection, compression of an aneurysm or stent graft, necrosis of the esophageal wall due to ischemia, and direct damage due to tumor growth or trauma. Since the AEF is lethal using conservative treatment [12], bleeding is often stopped by aortic stent graft insertion or descending aortic replacement [13]. Subsequently, we may perform bi-phasic esophagectomy and reconstruction, but postoperative management can also be agonizing [12]. There are no one-year survivors with AEFs after TEVAR without esophagectomy [14].

However, if the upper gastrointestinal bleeding is massive, we first stop bleeding using a specifically designed balloon tamponade device, such as the Minnesota Tube or SB tube [15,16]. Achieving hemostasis using an SB tube was effective in managing shock due to esophageal perforation of the descending aortic aneurysm [17]. Balloon tamponade devices are usually used for esophageal varices. Hemostatic efficacy is inadequate in AEFs, which is arterial high-pressure bleeding. Failure of hemostasis with balloon tamponade devices for esophageal varices due to cirrhosis has been reported in 35% of cases and adverse events in 20% [18]. Therefore, hemostatic failures and adverse events with balloon tamponade devices for arterial bleeding in AEFs are very high.　

In this case, the hemorrhagic shock caused by the AEF of an infected thoracic aortic aneurysm with dissection was treated with effective hemostasis using a Foley catheter. There are two reasons behind our ability to perform this life-saving intervention. First, we knew the location of the AEF based on the chest CT preoperatively. Second, hemostasis with tamponade was effective. In extreme emergencies, accurately placing a Foley catheter at the site of the fistula is difficult. In past reports, most catheters have been placed at the site of bleeding. In this case, the placement of the Foley catheter above the fistula was an important and novel finding because a Foley catheter could have strayed into the fistula at the site of bleeding and increased bleeding. Although there was no bleeding into the oral cavity at the beginning of the surgery, the anemia was progressive. A Foley catheter is available in most hospitals and ranges from small to large sizes. We selected and used it because physicians and nurses routinely use it on their patients for urine output measurements and know how to use it. We considered that blood was stored in the stomach from the site of Foley placement in the esophagus, which increased the internal pressure in the gastrointestinal tract and, thus, stopped the bleeding at the site of the fistula. Owing to the possible change in the balloon position, we considered the hemostatic effect to be temporary and prepared for bleeding. Normally, we would have used an SB tube. The SB tube must be used cautiously because the balloon is large. If inserted accidentally into a perforation site, there is a risk of enlargement of the perforation and additional complications. Therefore, in extreme emergencies, this technique can be difficult to use [19]. In addition, high balloon pressures risk compressing or damaging surrounding tissues. Prolonged implantation of a balloon tamponade device increases the risk of infection. If the balloon at the tip breaks, hemostasis may be inadequate, and the device must be reimplanted. Therefore, the placement of balloon tamponade devices requires technical skill and knowledge. We reaffirmed the importance of this classic therapy. Moreover, it is available in emergency situations where equipment and technology are limited, suggesting that its ease and flexibility may be useful. However, we must not forget that this is a blind process that should be paid careful attention to avoid serious complications.

## Conclusions

A patient with hemorrhagic shock caused by an AEF of an infected thoracic aortic aneurysm with dissection was successfully treated and saved using a Foley catheter. In similar cases, recognizing the location of the descending aorta and esophagus on preoperative imaging and preparing for the insertion of balloon tamponade devices are important, such as a Foley catheter or SB tube at any time during bleeding, depending on the situation. Moreover, achieving hemostasis using a Foley catheter, despite being an old technique, played an important role as a quick and effective first response and life-saving measure in the event of unexpected massive bleeding.
